# Safety and efficacy of minimally invasive percutaneous nephrolithotomy for infantile nephrolithiasis. Single centre experience from Pakistan

**DOI:** 10.3389/fped.2022.1035964

**Published:** 2023-01-16

**Authors:** Tariq Ahmad, Nasrum Minallah, Nida Khaliq, Hania Rashid, Misbah Syed, Moath Ahmad Abdullah Almuradi

**Affiliations:** ^1^ Department of Pediatric Urology, Institute of Kidney Diseases, Khyber Medical University, Peshawar, Pakistan; ^2^ Department of Urology, Institute of Kidney Diseases, Khyber Medical University, Peshawar, Pakistan; ^3^ Department of Community Medicine, Fazaia Medical Air University, Islamabad, Pakistan; ^4^ Department of Biochemistry, Fazaia Medical Air University, Islamabad, Pakistan; ^5^ Department of Urology, Khyber Teaching Hospital, Khyber Medical University, Peshawar, Pakistan

**Keywords:** infants, nephrolithiasis, percutaneous nephrolithotomy, PCNL, minimally invasive

## Abstract

**Objective:**

To assess the efficacy and safety of mini-percutaneous nephrolithotomy (PCNL) for small renal stones 1–2 cm in size in infants less than one year.

**Material and Methods:**

This descriptive case series was conducted in the department of pediatric urology Institute of Kidney Diseases Peshawar, Pakistan, from March 2019 to March 2022. All the patients underwent mini-PCNL in prone position under GA with 14 Fr access sheath and 10 Fr nephroscope. Stone clearance was assessed by non-contrast CT KUB at 30th postoperative day. Patients with no residual fragments on the non-contrast CT KUB were defined as stone-free. Patients with residual fragments of any size were defined as procedure failure. Safety was determined in terms of intra and postoperative complications.

**Results:**

A total of 51 infants were included in the study. The mean age of patients was 9.6 + 1.8 (5–12 month). The mean stone size was 15.8 + 2.7 (10–21) mm in length and 12.3 + 2.2 (8–17) mm in width. PCNL mean operative time was 51.6 ± 7.1 (40–70) minutes. Complete stone clearance at one month was observed in 46 (90.2%) patients. Residual fragments were seen in 5(9.8%) patients with a mean size of 1.6 + 0.4 (0.9–2.0) mm. None of the patients required any additional procedure for clearance of stones. In 7 (13.7%) patients, some post-operative complications were observe, all were grade I complications, including fever in 5(9.8%) and transient hematuria in 2(3.9%) patients.

**Conclusion:**

Mini-PCNL is a safe and effective treatment for renal stones in infants measuring 1–2 cm with high SFR and an acceptable complication rate.

## Introduction

The incidence of pediatric nephrolithiasis has increased over the last decade (1). Approximately 20% of urolithiasis cases in children have been observed in infants (2). This may be attributed to the increased awareness of the disease in the general population and the routine usage of ultrasonography (3). Risk factors for renal stones in infants include environmental and metabolic factors, anatomic anomalies, genetic or dietary factors and urinary tract infection (UTI) (4). The presenting symptoms of renal stones during infancy may include irritability, restlessness, UTIs, blood in urine, or passage of stones in urine (2, 4).

Flexible ureteroscopy (fURS), percutaneous nephrolithotomy (PCNL), and extracorporeal shock wave therapy (ESWL) are commonly used to treat renal stones in children. ESWL is usualy preferred for renal stones <2 cm in diameter (5). However, in children this modality requires general anesthesia (GA) and multiple sessions are needed for complete stone clearance (6). The potential for damage to growing kidneys has been demonstrated by a significant rise in the treated kidney's resistive index (RI) after ESWL (7). 49% of patients who received simultaneous bilateral ESWL for multiple stones or in repeated sessions were observed to have decreased glomerular filtration rates (GFRs) (8). PCNL is the benchmark for managing renal stones >2 cm in diameter. Although invasive, it provides a high stone free rate (SFR) as a monotherapy (5). However PCNL was not very much favoured in children due to the risks associated with treating smaller kidneys with larger instruments that may result in parenchymal damage and the associated impact on renal function (9). PCNL has been found not to cause adverse renal morphologic or functional alteration (10). Moreover, in the last few years, a lot of efforts have been made in terms of miniaturization of PCNL instruments, from standard PCNL (24–30 Fr) to mini-PCNL (18–22Fr), Ultramini-PCNL (11–14 Fr), and micro-PCNL (4.8 Fr) (11). This improvisation in instruments has not only assisted the surgical procedures to be used in the pediatric population but also made the possibility to treat the smaller stones that generally would be treated by ESWL.

Mini-PCNL is a feasible alternative to ESWL to treat small renal stones measuring 1–2 cm in size in children and infants. Only a few studies have been conducted on the safety and efficacy of mini-PCNL for managing renal stones in infants less than a year old. In this study, we aimed to show the efficacy and safety of mini-PCNL for small stones 1–2 cm in size in infants with age below one year.

## Materials and methods

This descriptive case series study was conducted after approval by the Institutional Review and Ethical Board (IREB) of the Institute of Kidney Diseases (IKD) at the department of pediatric urology IKD in Peshawar, Pakistan, from March 2019 to March 2022. Informed written consent was taken from the parents of all infants admitted for mini PCNL. We confirm that the study procedures fulfill the Declaration of Helsinki principles (12) and that the manuscript has been prepared according to STROBE guidelines (13).

Infants planned for mini-PCNL having normal renal functions, and negative urine cultures were included. Infants with solitary kidneys, ectopic kidneys, or horseshoe kidneys were excluded. All the patients underwent mini-PCNL. All the procedures were performed by the only pediatric urologist of the hospital. We recorded data including age, gender, renal stone characteristics, pre, and postoperative serum creatinine, pre and postoperative hemoglobin levels, per-operative data including cystoscopy, fluoroscopy and operative times, intra and postoperative complications, hospital stay, size of residual stones and SFR. Non-contrast CT KUB was performed to measure the stone size in milimeter. Postoperative Hb level was checked at 24 h postoperatively.

Efficacy was determined in terms of complete stone clearance. Stone clearance was assessed by non-contrast CT KUB at 30th postoperative day. Patients with no residual fragments on the non-contrast CT KUB were defined as stone-free. Patients with residual fragments of any size were defined as procedure failure. Residual stone fragments were graded based on their size: Grade-1: ≤2 mm, Grade-2: 2.1–4.0 mm, and grade-3: >4 mm. Safety was determined in terms of intra and postoperative complications. The intraoperative complications included failure of access, bleeding, colon injury, liver or splenic injury, renal pelvis injury, contrast extravasation, ureteropelvic junction injury and avulsion. Postoperative complications included fever (>38 °C), transient hematuria, need for blood transfusion, urine leakage and urosepsis. We defined the complications according to Clavien–Dindo system (14). The extracted stone fragments were sent to laboratory for analysis. Dietary advice and medical therapy were adequated postoperatively to all patients according to the composition of stones. We are following all patients with ultrasonography at six monthly intervals for stone recurrence and growth of the residual fragments.

Data were analyzed with SPSS version 25 software for windows. Frequencies and percentages were calculated for qualitative data, whereas means and standard deviations were calculated for quantitative data.

## Procedure

All the subjects were admitted to the unit a day before the procedure. Baseline investigations, including clotting profile, were performed. All children underwent mini-PCNL under GA. Cystourethroscopy was performed and 3 Fr ureteric catheter was passed in lithotomy position. Then, the position of the patient was changed into prone. Pelvicalyceal system (PCS) was opacified by retrograde administration of contrast (Urograffin). Puncture was made into the desired calyx by using an 18 G PCNL needle, under fluoroscopic guidance, using the Bull's Eye technique. The glide wire (0.035 inch) was passed through the needle into the PCS and ureter. The tract was dilated by using single step dilator, passed over the glide wire and secured with access sheath measuring 14 Fr wide and 8 cm long. It was followed by the introduction of 10 Fr nephroscope to reach the stone under direct vision. We used a manual irrigation system consisting of irrigation fluid connected to the nephroscope *via* an irrigation tube. We used normal saline as irrigant warmed to body temperature to avoid hyponatremia and hypothermia during the procedure. Stone fragmentation was done with pneumatic lithoclast (2 mm probe). Fragments were extracted using two prongs forceps. The decision to insert a double J stent (DJS) was individualized. We did not use nephrostomy in any of our patients. DJS was used if there was risk of ureteral blockage due to migration of stone fragments. DJS was removed four weeks after the procedure under GA.

## Results

The mean age of patients was 9.6 ± 1.8 (5–12 month). The stones were found in both right (*n* = 24) and left (*n* = 27) kidneys. The mean stone size was 15.8 ± 2.7 (10–21) mm in length and 12.3 ± 2.2 (8–17) mm in width with a mean stone density of 1,045 ± 237.6 (450–1,530) hounse field unit (HFU). The mean stone size and the demographics of the patients are listed in Table 1.

**Table 1:** Preoperative data of infants.

Variable	Result
Gender	
Male	30 (58.8%)
Female	21 (41.2%)
Age (months)	9.6 ± 1.8 (5–12)
Family History	
Yes	28 (54.9%)
No	23 (45.1%)
Stone Side	
Right	24 (47.1%)
Left	27 (5.9%)
Stone Size	
Length (mm)	15.8 ± 2.7 (10–21)
Width (mm)	12.3 ± 2.2 (8–17)
Guy's Stone Score	
1	42 (84.3%)
2	9 (17.6%)
3	0
4	0
Stone Density (HFU)	1045 ± 237.6 (450–1,530)
Stone Location	
Upper Pole	9 (17.6%)
Middle Pole	11 (21.6%)
Lower Pole	11 (21.6%)
Renal Pelvis	20 (39.2%)
Hemoglobin (gm/dl)	12.8 ± 1.1 (10.0–15.0)

All the PCNL procedures were carried out through one access tract. The procedure was performed by puncturing the most appropriate calyx. 59.9% were accessed through the middle pole calyx, 2% through the lower pole, whereas, 41.1% through the upper pole. Totally tubeless PCNL was performed on fifteen patients without any issue being faced during the procedure. DJS placement was carried out for thirty six patients. PCNL mean operative time was 51.6 ± 7.1 (40–70) minutes whereas mean time for fluoroscopy was 103.7 ± 11.9(80–120) seconds. The intraoperative data are shown in Table 2.

**Table 2:** Per-operative data of infants.

Variable	Result
Puncture Location	
Upper Pole	21 (41.1%)
Middle Pole	29 (56.9%)
Lower Pole	1 (2.0%)
Cystoscopy Time (min)	12.1 ± 2.0 (8–17)
Fluoroscopy Screen Time (sec)	103.7 ± 11.9 (80–120)
Operative Time (min)	51.6 ± 7.1 (40–70)
Complication	0
Stone Clearance by Fluoroscopy	100%
Double J Stent	
Yes	36 (70.6%)
No	15 (29.4%)

Complete stone clearance at one month was observed in 46 (90.2%) patients. Residual fragments were seen in 5(9.8%) patients with a mean size of 1.6 ± 0.4 (0.9–2.0) mm. None of the patients required any additional procedure for clearance of stones. No statistical difference was seen in post-operative and pre-operative serum creatinine levels (*p* > 0.05).

The mean hospital stay was 2.5 ± 0.8 (2–5) days. In 7 (13.7%) patients, some post-operative complications were observed. However, no death or serious complication was observed in our series; all were grade I complications, including fever in 5(9.8%) and transient hematuria in 2(3.9%) patients. No major complication such as hematuria requiring blood transfusion, urine leakage, or sepsis was observed in our study. Post-operative data are given in Table 3.

**Table 3:** Post-operative data of infants.

Variable	Result
Hemoglobin (gm/dl)	12.5 ± 1.0 (10–14.6)
Post-operative Complications	7 (13.7%)
Grade I	
Fever	5 (9.8%)
Transient Hematuria	2 (3.9%)
Grade II	0
Grade III	0
Grade IV	0
Grade V	0
Hospital Stay (days)	2.5 ± 0.8 (2–5)
Final Stone Clearance	
Yes	46 (90.2%)
No	5 (9.8%)
Residual fragment size (mm)	1.6 ± 0.4 (0.9–2.0)
Residual fragment grade	
Grade 1	5 (9.8%)
Grade 2	0
Grade 3	0

## Discussion

Newborns and infants who are diagnosed with renal stones during the first year of life usually have high chances of spontaneous resolution during the follow up. Andrioli et al. demonstrated that 75% of renal stones ≤5 mm in infants will resolve over a period of 1.1 year and only 15% will require surgical intervention. However stones >6 mm are less likely to resolve and will need surgical intervention. Other indications of surgery include symptomatic obstruction, sepsis and stone growth during the follow up (15). In the current study the renal stones were larger than 1 cm and symptomatic for which mini PCNL was performed.

The treatment of infantile nephrolithiasis is challenging due to the smaller size of the kidneys and the developing parenchyma. An efficient, safe, and less invasive surgical procedure followed by easy postoperative care is critical in treating these patients. The range of options available for treating renal stones in infants includes ESWL, fURS and PCNL.

ESWL in infants is performed under GA and requires repetitive sessions. In addition to the positioning difficulty that may occur, because of their smaller anatomy, the relative nearness of kidneys to lungs constitutes a higher risk for iatrogenic lung injury (16). Also, a large part of the infant's kidney will be exposed to increased energy, which theoretically can lead to a higher risk of acute renal damage, even though different studies revealed no long-term adverse effects of this exposure (17). Furthermore, residual stones after ESWL may lead to the recurrence of nephrolithiasis (18). In our institute ESWL is performed by technicians. Moreover anesthesia facility is also not available in the our ESWL suite. Pediatric URS has an excellent stone-free rate (SFR) with minimal intra or postoperative complications, given the standardized protocol-based approach (19). Flexible URS, however, requires LASER, which is not available in our part of the world. Besides, the procedure requires disposable and costly instruments, making it difficult to afford. The pelvicalyceal anatomy and narrow diameter of the ureter in infants can cause complications, such as ureteral injury, perforation, avulsion, ureteral stricture development, and pyelonephritis, an additional surgical intervention (20). Unfortunately we also don't have flexible URS and laser in our institute. Due to these limitations we favor mini PCNL over ESWL and fURS. The mini-PCNL procedure does not need disposable instruments and can safely be done with a pneumatic lithoclast. Mini-PCNL as monotherapy can lead to the best SFR compared to other minimally invasive techniques, such as ESWL and flexible URS (21).

The reported first session SFR for ESWL, flexible URS, and PCNL in infants are 53% (22), 87.4% (23) and 82.4% (24), respectively. To our knowledge this current study is the first study from Pakistan, reporting the outcomes of mini PCNL in infants, we achieved an SFR of 90.4%. We used a 14 Fr access sheath and 10 Fr nephroscope. Our SFR results are comparable to a study conducted in Bangalore in 2021, in which they reported their experience of minimally invasive PCNL in 24 infants, achieving an SFR of 91% (25). Our SFR results are higher than the study conducted by Dağgülli et al. and Bodakci et al. in which SFR results were 80% and 81.2%, respectively (18, 26). A similar study was conducted in USA by Jackman et al. who performed PCNL in eleven infants and preschool children using 11 Fr peel away sheath. They achieved complete stone clearance in 85% of the patient (27). However the mean age in their study was 3.4 years in comparison to 9.6 months in our study. Different studies were conducted in which they compared the SFR of mini-PCNL and ESWL for 1–2 cm stone in the pediatric population. The SFR after first session was 88.9% for PCNL and 55.6% for ESWL (*P* = 0.006) (28). Another study was done in China on 46 infants in which they compared the SFR of mini-PCNL and ESWL in infants under three years of age. The 1- and 3-month postoperative SFRs were 84% and 96% in the mini-PCNL group and 31.8 and 86.4% in the ESWL group (29). In 2019, a study in China reported their experience of micro-PCNL and flexible URS for renal stones in infants under two years of age. The SFR one month after surgery was 88.9% and 86.7%, respectively (30).

In multiple studies, the reported complication rates for ESWL, flexible URS, and mini-PCNL in children are 1.5%–35% (31), 10% (23) and 13.9% (32) respectively. In the present study, no intraoperative complication was observed. Postoperatively 13.7% of the patients developed only grade I complications, including fever in 9.8% and transient hematuria in 3.9%. None of our patients needed a blood transfusion. No case of urine leakage or sepsis was observed in the current study. The overall complication rate in our study is comparable to the study done in Bangalore by Patil et al., which is 16% (25). However, our complication rate is relatively better than that reported by Dağgülli et al., which was 40%, including bleeding requiring blood transfusion in 10%, fever in 10%, and UTI in 20% of the patients (18). Our complication rate is also better than that of Bodakci et al., which was 27%, including bleeding requiring blood transfusion in 4.2%, fever in 8.3%, and UTI in 14.6% of the patients (26).

The cause of renal stones in infants is uasually a metabolic disorder. Therefore full metabolic evaluation should be perfomed in these patiients. Dietary and medical therapy should be initiated according to the stone compostion to prevent stone recurrence. These patients, especialy those with residual fragments need regular follow up to monitor the progression of the disease.

The study's limitations may include the associated risk of radiation exposure to infants during the procedure. Other limitations are the small sample size, experience from a single center, and the study's observational nature.

## Conclusion

We concluded that mini-PCNL is a safe and effective treatment for renal stones in infants measuring 1–2 cm with high SFR and an acceptable complication rate. However, further studies with a large sample size from multiple centers and randomized control trials should be conducted to further probe into the outcomes of this procedure in infants for 1–2 cm renal stones.

## Data Availability

The raw data supporting the conclusions of this article will be made available by the authors, without undue reservation.

## References

[1] Çamlar SA Soylu A Kavukçu S. Characteristics of infant urolithiasis: a single center experience in western Turkey. J Pediatr Urol. (2020) 16(4):463.e1-.e6. 10.1016/j.jpurol.2020.05.005 32571536

[2] Baştuğ F Gündüz Z Tülpar S Poyrazoğlu H Düşünsel R. Urolithiasis in infants: evaluation of risk factors. World J Urol. (2013) 31(5):1117–22. 10.1007/s00345-012-0828-y 22258667

[3] Yilmaz K Dorterler ME. Characteristics of presentation and metabolic risk factors in relation to extent of involvement in infants with nephrolithiasis. Eurasian J Med. Invest. (2020) 4:78–85. 10.14744/ejmi.2019.87741

[4] Alemzadeh-Ansari MH Valavi E Ahmadzadeh A. Predisposing factors for infantile urinary calculus in south-west of Iran. Iran J Kidney Dis. (2014) 8(1):53–7. 24413722

[5] Tekgül S Riedmiller H Gerharz E Hoebeke P Kocvara R Nijman R Guidelines on paediatric urology. (2015)13–5.

[6] Alsagheer G Abdel-Kader MS Hasan AM Mahmoud O Mohamed O Fathi A Extracorporeal shock wave lithotripsy (eswl) monotherapy in children: predictors of successful outcome. J Pediatr Urol. (2017) 13(5):515.e1-.e5. 10.1016/j.jpurol.2017.03.029 28457667

[7] Aoki Y Ishitoya S Okubo K Okada T Maekawa S Maeda H Changes in resistive index following extracorporeal shock wave lithotripsy. Int J Urol. (1999) 6(10):483–92. 10.1046/j.1442-2042.1999.00097.x 10533899

[8] Cass AS. Renal function after bilateral extracorporeal shockwave lithotripsy. J Endourol. (1994) 8(6):395–9. 10.1089/end.1994.8.395 7703989

[9] Etemadian M Maghsoudi R Shadpour P Mokhtari MR Rezaeimehr B Shati M. Pediatric percutaneous nephrolithotomy using adult sized instruments: our experience. Urol J. (2012) 9(2):465–71. 10.22037/uj.v9i2.1473 22641489

[10] Wadhwa P Aron M Bal CS Dhanpatty B Gupta NP. Critical prospective appraisal of renal morphology and function in children undergoing shockwave lithotripsy and percutaneous nephrolithotomy. J Endourol. (2007) 21(9):961–6. 10.1089/end.2006.9928 17941769

[11] Sultan S Aba Umer S Ahmed B Naqvi SAA Rizvi SAH. Update on surgical management of pediatric urolithiasis. Front Pediatr. (2019) 7:252. 10.3389/fped.2019.00252 31334207 PMC6616131

[12] World medical association declaration of helsinki. Ethical principles for medical research involving human subjects. Bull World Health Organ. (2001) 79(4):373–4. 11357217 PMC2566407

[13] von Elm E Altman DG Egger M Pocock SJ Gøtzsche PC Vandenbroucke JP. The strengthening the reporting of observational studies in epidemiology (strobe) statement: guidelines for reporting observational studies. Int J Surg. (2014) 12(12):1495–9. 10.1016/j.ijsu.2014.07.013 25046131

[14] Clavien PA Barkun J de Oliveira ML Vauthey JN Dindo D Schulick RD The clavien-dindo classification of surgical complications: five-year experience. Ann Surg. (2009) 250(2):187–96. 10.1097/SLA.0b013e3181b13ca2 19638912

[15] Andrioli V Highmore K Leonard MP Guerra LA Tang K Vethamuthu J Infant nephrolithiasis and nephrocalcinosis: natural history and predictors of surgical intervention. J Pediatr Urol. (2017) 13(4):355.e1-.e6. 10.1016/j.jpurol.2017.06.010 28729176

[16] McLorie GA Pugach J Pode D Denstedt J Bagli D Meretyk S Safety and efficacy of extracorporeal shock wave lithotripsy in infants. Can J Urol. (2003) 10(6):2051–5. 14704109

[17] El-Nahas AR Awad BA El-Assmy AM Abou El-Ghar ME Eraky I El-Kenawy MR Are there long-term effects of extracorporeal shockwave lithotripsy in paediatric patients? BJU Int. (2013) 111(4):666–71. 10.1111/j.1464-410X.2012.11420.x 22924860

[18] Dağgülli M Sancaktutar AA Dede O Utanğaç MM Bodakçi MN Penbegül N Minimally invasive percutaneous nephrolithotomy: an effective treatment for kidney stones in infants under 1 year of age. A single-center experience. Urolithiasis. (2015) 43(6):507–12. 10.1007/s00240-015-0787-z 26002160

[19] Jones P Rob S Griffin S Somani BK. Outcomes of ureteroscopy (urs) for stone disease in the paediatric population: results of over 100 urs procedures from a UK tertiary centre. World J Urol. (2020) 38(1):213–8. 10.1007/s00345-019-02745-3 30949802 PMC6954136

[20] Resorlu B Oguz U Resorlu EB Oztuna D Unsal A. The impact of pelvicaliceal anatomy on the success of retrograde intrarenal surgery in patients with lower pole renal stones. Urology. (2012) 79(1):61–6. 10.1016/j.urology.2011.06.031 21855968

[21] Kallidonis P Tsaturyan A Lattarulo M Liatsikos E. Minimally invasive percutaneous nephrolithotomy (pcnl): techniques and outcomes. Turk J Urol. (2020) 46(Supp. 1):S58–s63. 10.5152/tud.2020.20161 32525477 PMC7731950

[22] Lottmann HB Archambaud F Traxer O Mercier-Pageyral B Helal B. The efficacy and parenchymal consequences of extracorporeal shock wave lithotripsy in infants. BJU Int. (2000) 85(3):311–5. 10.1046/j.1464-410x.2000.00475.x 10671889

[23] Ishii H Griffin S Somani BK. Ureteroscopy for stone disease in the paediatric population: a systematic review. BJU Int. (2015) 115(6):867–73. 10.1111/bju.12927 25203925

[24] Penbegül N Tepeler A Sancaktutar AA Bozkurt Y Atar M Yildirim K Safety and efficacy of ultrasound-guided percutaneous nephrolithotomy for treatment of urinary stone disease in children. Urology. (2012) 79(5):1015–9. 10.1016/j.urology.2011.10.059 22196411

[25] Patil N Javali T Hamsa V Nagaraj HK. A single centre experience of percutaneous nephrolithotomy in infants and its long-term outcomes. J Pediatr Urol. (2021) 17(5):650.e1-.e9. 10.1016/j.jpurol.2021.07.026 34417130

[26] Bodakci MN Daggülli M Sancaktutar AA Söylemez H Hatipoglu NK Utangaç MM Minipercutaneous nephrolithotomy in infants: a single-center experience in an endemic region in Turkey. Urolithiasis. (2014) 42(5):427–33. 10.1007/s00240-014-0677-9 25004801

[27] Jackman SV Hedican SP Peters CA Docimo SG. Percutaneous nephrolithotomy in infants and preschool age children: experience with a new technique. Urology. (1998) 52(4):697–701. 10.1016/s0090-4295(98)00315-x 9763096

[28] Farouk A Tawfick A Shoeb M Mahmoud MA Mostafa DE Hasan M Is mini-percutaneous nephrolithotomy a safe alternative to extracorporeal shockwave lithotripsy in pediatric age group in borderline stones? A randomized prospective study. World J Urol. (2018) 36(7):1139–47. 10.1007/s00345-018-2231-9 29450731

[29] Zeng G Jia J Zhao Z Wu W Zhao Z Zhong W. Treatment of renal stones in infants: comparing extracorporeal shock wave lithotripsy and mini-percutaneous nephrolithotomy. Urol Res. (2012) 40(5):599–603. 10.1007/s00240-012-0478-y 22580634

[30] Wang W Beijing JL. Mp79-01 comparing micro-percutaneous nephrolithotomy and flexible ureteroscopic lithotripsy in treating 1-2 cm solitary renal stones in infants. J Urol. (2019);201(Supplement 4): e1151–e2. 10.1097/01.JU.0000557353.37272.21

[31] Slavkovic A Radovanovic M Vlajkovic M Novakovic D Djordjevic N Stefanovic V. Extracorporeal shock wave lithotripsy in the management of pediatric urolithiasis. Urol Res. (2006) 34(5):315–20. 10.1007/s00240-006-0062-4 16868754

[32] Jones P Bennett G Aboumarzouk OM Griffin S Somani BK. Role of minimally invasive percutaneous nephrolithotomy techniques-micro and ultra-mini pcnl (<15f) in the pediatric population: a systematic review. J Endourol. (2017) 31(9):816–24. 10.1089/end.2017.0136 28478724

